# Iron-Deficiency in Atopic Diseases: Innate Immune Priming by Allergens and Siderophores

**DOI:** 10.3389/falgy.2022.859922

**Published:** 2022-05-10

**Authors:** Franziska Roth-Walter

**Affiliations:** ^1^Comparative Medicine, The Interuniversity Messerli Research Institute, University of Veterinary Medicine Vienna, Medical University Vienna, University of Vienna, Vienna, Austria; ^2^Institute of Pathophysiology and Allergy Research, Center of Pathophysiology, Infectiology and Immunology, Medical University of Vienna, Vienna, Austria

**Keywords:** iron-deficiency, atopic diseases, pathogenesis-related proteins, siderophores, polyphenols, lipocalin, holoBLG, immunonutrition

## Abstract

Although iron is one of the most abundant elements on earth, about a third of the world's population are affected by iron deficiency. Main drivers of iron deficiency are beside the chronic lack of dietary iron, a hampered uptake machinery as a result of immune activation. Macrophages are the principal cells distributing iron in the human body with their iron restriction skewing these cells to a more pro-inflammatory state. Consequently, iron deficiency has a pronounced impact on immune cells, favoring Th2-cell survival, immunoglobulin class switching and primes mast cells for degranulation. Iron deficiency during pregnancy increases the risk of atopic diseases in children, while both children and adults with allergy are more likely to have anemia. In contrast, an improved iron status seems to protect against allergy development. Here, the most important interconnections between iron metabolism and allergies, the effect of iron deprivation on distinct immune cell types, as well as the pathophysiology in atopic diseases are summarized. Although the main focus will be humans, we also compare them with innate defense and iron sequestration strategies of microbes, given, particularly, attention to catechol-siderophores. Similarly, the defense and nutritional strategies in plants with their inducible systemic acquired resistance by salicylic acid, which further leads to synthesis of flavonoids as well as pathogenesis-related proteins, will be elaborated as both are very important for understanding the etiology of allergic diseases. Many allergens, such as lipocalins and the pathogenesis-related proteins, are able to bind iron and either deprive or supply iron to immune cells. Thus, a locally induced iron deficiency will result in immune activation and allergic sensitization. However, the same proteins such as the whey protein beta-lactoglobulin can also transport this precious micronutrient to the host immune cells (holoBLG) and hinder their activation, promoting tolerance and protecting against allergy. Since 2019, several clinical trials have also been conducted in allergic subjects using holoBLG as a food for special medical purposes, leading to a reduction in the allergic symptom burden. Supplementation with nutrient-carrying lipocalin proteins can circumvent the mucosal block and nourish selectively immune cells, therefore representing a new dietary and causative approach to compensate for functional iron deficiency in allergy sufferers.

## Introduction

The ability of iron to act as an electron receptor or donor forms the fundamental basis for its essential role in supporting basic cellular processes, of which oxygen transport *via* iron-containing heme in hemoglobin is the most well-known (1). As such, iron is not only essential for humans but extends to almost all organisms that we consume (e.g., plants, animals), symbiotically live with as commensal microbes or are pathogenic and infect us.

Although iron is one of the most common elements on earth, about a third of the world's population are affected by iron deficiency, with, predominantly, infants, preschool children, young menstruating women, and women in the second/third trimester of pregnancy and postpartum being affected (2, 3). In western countries, female gender and persons with a vegetarian or vegan diet, blood donors but also elite endurance athletes due to inflammation-induced functional iron deficiency are at greater risk (4).

Besides blood loss, there are two main drivers for iron deficiency, chronic lack of dietary iron, and/or a hampered uptake machinery usually as a result of immune activation. Iron is closely linked with our immune system as the major contributor for systematic iron recycling; shuttling and distribution are the macrophages, which are also key cells in innate immunity, with their iron status determining activation or suppression of the immune machinery.

Many respiratory allergens, such as pathogenesis-related proteins and lipocalins, are able to deprive antigen-presenting cells from iron, thereby initiating presentation and immune activation. Iron deficiency also favors survival of Th2-cells, facilitates antibody class switching, and is also an essential contributor in the effector phase as a lack of iron primes mast cells for degranulation.

In this review, we highlight the most important interconnections between iron metabolism and allergies, the effect of iron deprivation on distinct immune cell types, as well as the pathophysiology in atopic diseases. Although the main focus will be humans, we also compare them with innate defense and iron sequestration strategies of microbes and plants important for the etiology of allergic diseases and give epidemiology, preclinical and clinical evidence for exploiting the iron-immune regulatory axis to combat the atopic march.

## Basic Iron Features

Iron is present in our body mainly in the ferrous (Fe2+, acting as an electron donor) or ferric form (Fe3+, an electron acceptor). Under anaerobic conditions, the ferrous form, which preferentially binds to nitrogen and sulfur ligands (5), is favored, whereas, in oxygen-rich environments, ferric iron is the most dominant form. Due to its incredible high affinity to oxygen, “free iron” is biochemically dangerous as it can damage tissue by catalyzing the formation of oxygen radicals that attack cellular membranes, proteins, and DNA (1) (Haber-Weiss reaction). Hence, under healthy conditions, no appreciable concentration of “free iron” is present as iron is virtually always present in a complexed form (e.g., as heme) and/or protein-bound form (e.g., bound to transferrin, lactoferrin, etc.) (6). Moreover, iron uptake is highly regulated with a sophisticated iron-uptake machinery existing not only in humans (7) but also in bacteria (8), fungi, and plants (9), emphasizing that iron acquisition is always an active, regulated process.

## Non-Transferrin Bound Iron and the Labile Iron Pool

The non-transferrin bound iron pool (NTBI) represents the presence of iron, not bound by transferrin in the circulation. As such, it comprises the ferric iron-binding proteins lactoferrin and ceruloplasmin, a copper-containing ferroxidase that is essential to export iron out from the tissue to the circulation. It includes members of the lipocalin family, such as LCN1 and LCN2 (10–12), binding to a plethora of iron-siderophore complexes but also to heme as the lipocalin alpha1-microglobulin (13–16). Moreover, heme-binding proteins, such as hemopexin and peroxynitrite isomerase THAP4 (17), as well as haptoglobulin binding to heme-containing hemoglobin and a large number of poorly defined low molecular weight, belong to the NTBI. Known low-molecular weight compounds of the NTBI are ferric iron-binding citric acid, being the major representative here (18) but extending to amino acids, such as glycine and asparagine (19), ATP/AMP, and catecholamines [dopamine (20), norepinephrine (21), and epinephrine (22)]. Dietary-derived catechol flavonoids have also been suggested to be part of the NTBI that partake in iron homeostasis (23).

Intracellularly, iron concentration is about 1 μM but may range from 0.5 to 10 μM (24, 25) and is part of the so-called labile iron pool, LIP, for further incorporation into iron-dependent enzymes and electron transfer proteins, with glutathione acting presumably as a cellular buffer (26). The ferritin H subunit (FTH) oxidizes ferrous to ferric iron for storage within ferritin. Although the ferrous form seems to be intracellular prevalent, endogenous ferric-binding siderophore such as 2,5-dihydroxybenzoic acid (26) also partakes in iron transport and homeostasis (26), with a deficiency here causing intracellular iron accumulation.

## Iron Status in the Steady State

The human body contains about 4-to-5-g iron with men having, on average, 50 mg/kg and women about 38 mg/kg. Roughly, two thirds of the total body iron is contained in heme within hemoglobins in red blood cells (27), with the next biggest store being the liver (≈1 g) and the mononuclear phagocyte system (≈0.6 g), in which iron is stored in ferritin (28) as ferrihydrates and in hemosiderin, which is a poorly defined iron-storage complex, presumably composed of ferritin, denatured ferritin, and other materials (29). About 0.3 g of iron in heme is present in the myoglobins of the muscles (30, 31). All other cellular iron-containing proteins and enzymes are estimated to bind a total of about 8 mg of iron.

### Dietary Iron Uptake

The daily uptake of iron through food is about 1–2 mg, just as high as the daily loss of iron through desquamation of the enterocytes lining the gut or of the skin and due to smaller bleedings. Iron may leave the body also through urine, bile or sweat, although in considerable smaller and usually neglectable amounts (32–34).

About 10–20 mg iron is consumed daily *via* the normal diet representing the major iron source in humans, of which a tenth is absorbed. Within the digestive tract, iron is present in two forms: as heme iron (meat, fish) and non-heme iron (cocoa, legumes, cereals, fruits) of which heme-iron uptake is about five times more efficiently absorbed than non-heme iron. Its bioavailability is further determined by the individual iron status and physiological condition and is reflected by the production of hepcidin (35).

The chief area of iron absorption is the duodenum and the proximal jejunum (36), which is more acidic, with a pH ranging from 4 to 5 than the rest of small intestines, with a pH range between 7 and 9. It is also the site where pancreatic juices and bile enter the small intestines.

Heme iron is transported as heme (from meat) into the enterocytes *via* the known transporter for folate being the high-affinity folate transporter PCP/HCP1 (SLC46A1) (37–39), and also the duodenal cytochrome b; Dcytb is able to bind on the lumen and on the cytoplasmic side to heme molecules (40–44).

**Figure 1:**
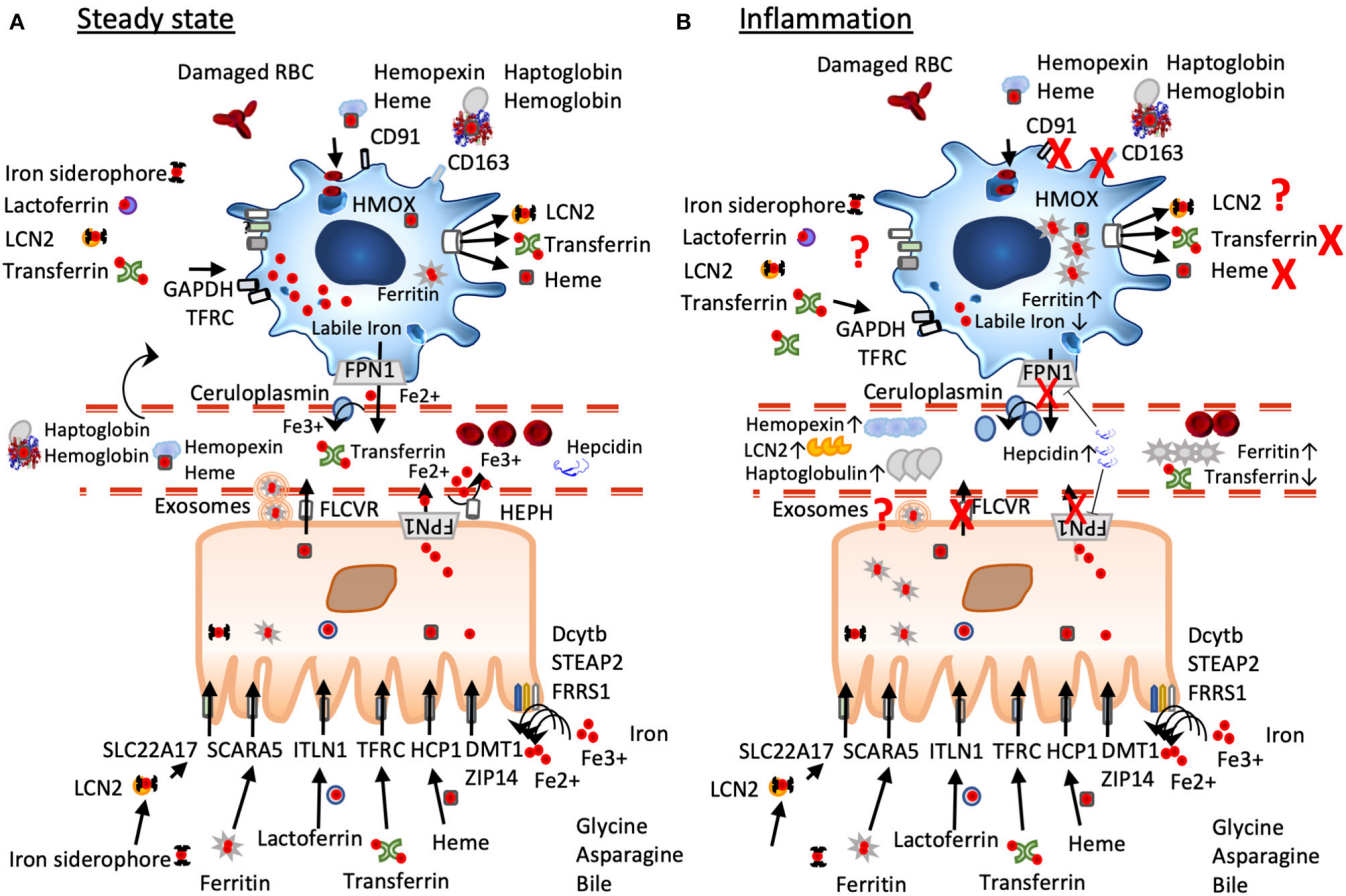
A simplified scheme of iron homeostasis under steady-state and inflammatory conditions. **(A)** Under non-inflamed steady-state conditions, iron is reduced by ferric reductases (Dcytb, STEAP2, FRRS1) in the intestinal lumen to ferrous iron before import *via* DMT1 and ZIP14, heme iron is transported *via* the folate receptor HCP1, lactoferrin *via* ITLN1, dietary ferritin uptake occurs *via* SCARA 5, and chelated iron can be captured by LCN2 and transported by the enterocytes *via* SLC22A17. Cellular iron export occurs *via* ferroportin often aided by hephaestin and/or ceruloplasmin, ferritin seems to be exported *via* exosomal pathways, heme is exported *via* FLCVR. Macrophages under steady state have an anti-inflammatory phenotype characterized by a large labile iron pool, low ferritin-levels, and expression of iron importers such as CD163. They constantly take up but also export iron that derives from damaged red blood cells, from heme-hemopexin, haptoglobin-hemoglobin, LCN2, transferrin, and lactoferrin. **(B)** Under inflammation, iron mobilization is blocked due to increased expression of hepcidin that leads to FPN degradation and trapping iron inside the cells. Macrophages change to an inflammatory phenotype inhibiting iron import and export, their ferritin-levels are increased, while their labile iron pool is decreased. In the circulation levels of ferritin, hemopexin, haptoglobulin, and lipocalin 2 are elevated, while serum iron and transferrin are decreased.

For non-heme iron, which is typically ferric iron chelated by low molecular weight compounds (e.g., plants, meat), reduction by ascorbic acid and/or duodenal ferric reductases, such as cytochrome b, Dcytb, STEAP2, and FRRS1 (41, 42), has to precede before uptake *via* the divalent metal-ion transporter 1, DMT1, and ZIP14 is initiated (44, 45). Iron-carrying proteins, such as lactoferrin (46), transferrin (47), or ferritin from food, are efficiently absorbed without depending on reduction or heme transporter *via* receptor-mediated, clathrin-dependent endocytosis: ferritin *via* SCARA5 (48), lactoferrin *via* ITLN1 (49). Moreover, glycine and asparagine, but not other amino acids (19), promote iron absorption (50) (Figure 1).

Iron can also be transported *via* the lymphatic system, with bile itself contributing to iron absorption (51–53). Newer dietary iron-supplementation formulation encapsules iron [ferrous iron (54)] with a phospholipid bilayer generating a liposomal iron or surround ferric iron in sucrosomes (starchlike vesicles) (55), which leads to uptake of iron *via* the lymphatic system and circumvent hepcidin-mediated blockage of iron absorption (56).

Once in the cell, iron is exported *via* the iron exporter ferroportin 1 (IREG1, MTP1, SLC40A1, FPN1, HFE4) (57), often with the help of Hephaestin HEPH or ceruloplasmin CP and is released into the circulation. Ferroportin-mediated iron efflux is calcium activated and functions as an iron/calcium antiporter (58).

Heme iron export occurs *via* the Feline leukaemic virus receptor (FLVCR) (59, 60), which is also highly expressed in enterocytes, and is dependent on hemopexin (61, 62). Ferritin seems to be exported *via* exosomes (63) (Figure 1). In general, iron excretion is suppressed by inflammation and enhanced during erythropoiesis and hypoxia (44).

Dietary phytates, representing inositol polyphosphates typically contained in nuts, seeds, and grains, form insoluble precipitates with iron (64) and thus inhibit dietary uptake (65). Similarly, fruit- and plant-derived polyphenolic compounds are known to reduce the bioavailability for non-heme iron as many of these bind with high affinity to iron (66). Upon consumption, flavonoid concentrations in plasma can reach 1–10 μM (67) and thus may considerably influence iron homeostasis (68, 69). Consequently, consumption of large quantities of purified polyphenols has been reported to decrease the volunteers' iron status (70–73). However, when these polyphenols are already in complex with iron, dietary administration of polyphenol-iron complexes had been demonstrated to contribute to an improved iron and redox status *in vivo* (74, 75).

### Iron Regulation

In 2001, hepcidin, which is highly conserved between species and only 25-amino acids long, was discovered as the key regulator for systemic iron homeostasis (76). It is mainly secreted by the liver in response to iron overload or inflammation (77), but, also, parietal cells of the stomach (78) and macrophages synthesize and secrete hepcidin. Under steady state, hepcidin is found in the plasma in a protein-bound and free-circulating form (79), with only the latter being excreted into the urine (80). Reported hepcidin concentration in the circulation is about 7.8 nM in men, 4.1 nM in pre-, and 8.5 nM in post-menopausal women (81). Radiolabeled hepcidin accumulated in the ferroportin-rich organs, liver, spleen, and proximal duodenum (82).

Hepcidin decreases plasma iron levels by blocking iron absorption in the duodenum and iron release from macrophages, thus targeting the two entrance gates for iron into the circulation. Molecularly, it binds to ferroportin (FPN), inducing its internalization, ubiquitinylation, and consecutive degradation of FPN in the lysoproteasome (77), while iron is retained within the cells (81, 83). Hepcidin is also negatively regulated by folic acid, cobalamin, or vitamin D (84).

Under iron-replete conditions, increasing body iron levels cause an increased hepcidin expression, hampering further iron accumulation and acquisition in macrophage and liver cells, and decreased dietary iron absorption; the result is a reduction in serum iron (85). In contrast, when more iron is needed, hepcidin decreases, permitting macrophages to release iron and allowing an enhance uptake of dietary iron via the gut.

As hepcidin is also an acute phase reactant, it is upregulated during inflammation to remove iron from the circulation along with iron-binding proteins, such as lactoferrin, haptoglobulin, hemopexin, lipocalin 2, and ferritin (81, 86). Due to its dual role in iron regulation and inflammation, hepcidin levels in the circulation reflect on the one hand ongoing inflammation as well as the need of iron; consequently, in conditions of severe anemia and inflammation, low hepcidin levels will prevail despite the presence of inflammation (87).

### Iron in the Circulation

Iron is then delivered to most tissues *via* circulating transferrin, which carries roughly 2 mg of this metal in the steady state (88). Hemopexin also seems to partake in distributing dietary heme iron, which accounts for two-thirds of absorbed body iron, as a lack of hemopexin leads to heme accumulation in the enterocyte and impedes heme distribution (89). In healthy men, plasma iron turnover ranges from 25 to 35 mg (90) per day, of which only 5 to 10% is provided by absorption of dietary iron in the gut, the rest being predominantly iron recycled from monocytes and macrophages of the liver, adipose tissue, bone marrow, spleen, and lymph nodes (91). Regarding serum levels, most iron-associated proteins dedicated to distributing and mobilizing iron are increased in situations of greater iron demand such as transferrin, hemopexin, soluble transferrin receptor, and ceruloplasmin (92, 93), while serum iron is low. In contrast, reduced levels of the same proteins in the serum/plasma at steady-state condition usually describe the consequence of an effective iron delivery to the target tissues (e.g., transferrin-iron binding to transferrin receptor 1 CD71, heme-hemopexin complex binding to CD91 expressed on hepatocytes, monocytes, and macrophages in the spleen and liver, haptoglobulin-hemoglobin binding on CD163 expressed on M2-macrophages) and indicate an improved iron status.

In contrast to the widely disturbed transferrin receptor 1 TFRC responsible for iron import *via* iron-sated transferrin, transferrin receptor 2 (373) (mainly expressed by hepatocytes, erythroid cells, but also by basophils and eosinophils) bind to erythropoietin (94, 372), exert a regulatory function (95) and do not participate in increasing tissue iron. Ablation or mutation of this receptor leads to iron overload (95, 96) in the respected tissue.

## Iron Deficiency in Humans

As iron homeostasis is quite complex, there is still no international consensus that clearly defines iron deficiency (97) with the World Health Organization (WHO) defining anemia as circulating hemoglobin (Hb) levels <12. g/dL in non-pregnant women and <13. g/dL in men (98, 99). However, normal Hb distribution varies not only with sex but also with ethnicity and physiological status; thus, recommended adjustment factors are given by the WHO according to, e.g., smoking habits and people living above 1,000-m altitude (100). Ferritin is a good indicator for iron stores, but also, here, adjustments are done (101) and recommended as ferritin is elevated upon infection or inflammation (102). Thus, the assessment of the iron status is not precise, since the available biomarkers reflect the iron status of different compartments in the body: serum ferritin assesses stored iron, while serum iron and the percentage of transferrin saturation reflect the iron supply to tissues. Serum transferrin receptor, erythrocyte ferritin, and red cell zinc protoporphyrin are indicators for the iron supply to the bone marrow, whereas the percentage of hypochromic red blood cells, mean corpuscular volume, and reticulocyte hemoglobin reflect the use of iron by the bone marrow. As these biomarkers are affected by age, sex, disease (infections, inflammation), life style (e.g., blood donations, smoking, drugs, physical fitness), there is currently no single standardized test that can diagnose iron deficiency without anemia, and even the use of multiple tests can only partially overcome the limitations of individual tests, especially because many iron markers are elevated during inflammatory responses or mild immune activation (103).

According to the Global Burden of Disease Study 2016, estimated 1.24 billion individuals are affected by iron deficiency anemia, with the figures for the global prevalence of iron deficiency without anemia being estimated at least double.

Immune activation and iron balance are intertwined, with a change in the iron status always modulating the immunological reactivity. This is reflected in the two main entities of iron deficiency being anemia and “functional iron deficiency.” However, various shades and mixed forms between these two are possible. During functional iron deficiency, iron is not “mobilized,” leading to functional impairments of cells and tissues. Only in severe cases, this results in anemia, which represents the most extreme example of iron deficiency. In mild to moderate cases of iron deficiency, anemia is not present, although the function of tissues and cells is already compromised.

Virtually, every immune activation results in functional iron deficiency (4, 104–108), where, despite sufficient iron stores in the liver and mononuclear phagocyte system (macrophages), iron mobilization is inhibited and dietary iron absorption is decreased by hepcidin, the master regulator of iron uptake. As such, even in healthy adults, iron deficiency is a driver of low-grade chronic inflammation (109).

Persons with functional iron deficiencies usually suffer from underlying chronic or metabolic diseases such as autoimmune (110, 111) and atopic diseases (108, 112–115), chronic kidney diseases (56, 116, 117), congestive heart failure (118–120), chronic pulmonary diseases (121–123), and obesity (124, 125), in which iron deficiency is associated with a worsened prognosis and outcome (103, 104, 126–133). Interestingly, iron deficiency is also associated with an increased risk for thrombosis (134, 135).

## Iron Recycling by Macrophages—the Direct Link to our Immune System

As duodenal dietary iron uptake only accounts for 1–2 mg of the daily acquirements, iron is recycled largely through the erythrocyte hemoglobin cycle as the novo synthesis of hemoglobin consumes about 25 mg iron per day. Iron is recycled from senescent red blood cells by macrophages. Recycling occurs predominantly in the spleen by the for this purpose specialised red pulp macrophages and to a lesser degree also Kupfer cells in the liver can recycle iron from red blood cells. Both macrophage-types in the splenic red pulp as well as in the liver have by default an anti-inflammatory phenotype and are critical for maintaining systemic iron concentration (130).

Macrophages are the principal cells responsible for handling iron in mammals, and, thus, any change in the iron status has a direct impact on the innate and, indirectly, on the adaptive immune system.

Macrophages are present in all tissues and classically appreciated for their surveillance role in pathogen recognition. They have crucial homeostatic function, including cell repair, phagocytic clearance of apoptotic and senescent cells, and even cell death. Moreover, in the last decade, their function to support and restore the tissue homeostatic balance, by acting, on the one hand, as sensors for the local iron demands and, on the other hand, providing the local environment with the essential trace element iron, became apparent (130).

**Figure 2:**
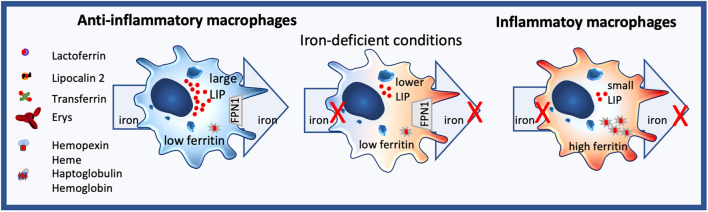
Iron homeostasis in macrophages. Anti-inflammatory macrophages constantly take up but also export iron and are characterized by a large labile iron pool (LIP) and low ferritin levels. In contrast, inflammatory macrophages neither import nor efflux iron, their LIP is small, while ferritin expression is high. Under iron-deficient conditions, no iron can be distributed by anti-inflammatory macrophages, changing their phenotype towards a more inflammatory state.

Macrophages are sentinels, who are highly plastic, and whole spectra of macrophage subtypes and activation status exist, ranging from an M1-like proinflammatory to an M2-like tissue repair phenotype. Importantly, they markedly differ in their iron handling (136). Indeed, M2 macrophages usually express highly CD163, the hemoglobin/haptoglobin receptor, have low ferritin levels, while having a large labile iron pool LIP, and the iron-export protein, ferroportin FPN, is highly expressed (Figure 2). In contrast, M1 macrophages do not partake in iron sequestration, although they favor an iron storage phenotype having a low LIP, increased ferritin-levels and decreased FPN expression (Figure 2) (126, 137, 138).

Of note, in the healthy steady-state conditions, the increased iron uptake by phagocytosis of senescent red blood cells, uptake of hemoglobin (139, 140), hemoglobin-haptoglobin complexes (141, 142), heme-hemopexin (143–145), iron-siderophore laden lipocalin 2 (LCN2) (146–150), iron-laden ferritin (138, 151–155) does not induce inflammation, but, rather, contrarily promotes an anti-inflammatory macrophage phenotype and thus contributes to immune suppression, regulation, and restoration of the tissue homeostatic function as, simultaneously, they serve as iron-rich nurse cells supporting other cells and tissues with iron (148).

In line, macrophage-derived transferrin has been shown to contain already iron and supports lymphocyte proliferation (156).

## Atopic Diseases and Micronutrients

The tendency to develop allergies, also called atopy, affects almost one third of the Western population and is partly inherited. Especially in our affluent society, the development of allergy is paradoxically characterized by a lack of contacts and the absence of micronutrients.

On the one hand, the lack of contact with people, animals, and germs leaves the immune system untrained, and, thus, several deficiencies of innate proteins, such as LCN2 (157), lactoferrin (158), uteroglobin (SCGB1A1) (159), Cathelicidin antimicrobial peptide (160), have been described in atopic individuals compared to non-allergic ones, which further underline the lack of microbial contact but also the lack of nutritional support by commensal microbes in atopic individuals.

On the other hand, a lack of micronutrients signals danger to the immune cells and often leads—through this heightened alertness—to an exaggerated immune response, which is such a typical characteristic in individuals with allergy (161, 162). Due to the heightened immune response, patients with atopic diseases also have an increased risk to develop autoimmune diseases (113).

In contrast, studies reveal that the earlier children have contact with other children, as well as animals, the less likely they are suffering from allergies (163). The probability of developing an allergy decreases with the number of siblings and the ownership of pets (164), for example, dogs, and it is proven that regular stays in the immediate vicinity of farms protect against the development of asthma and hay fever (165).

### Micronutritional Deficiencies in Atopic Individuals

Especially in the perinatal period, an adequate nutrition is pivotal to avoid an atopic predisposition (166, 167). A plethora of studies affirm that atopics suffer from numerous micronutrient deficiencies (114, 115, 168–180), such as vitamins A (181), E, (182, 183), and D, as well as folic acid and iron (112, 162). Although usually widely overlooked, these micronutrients have a profound impact on our genes and our immune system, resulting in many epigenetic changes affecting immune-associated genes (167, 184), but, most importantly, being also associated with enhanced inflammatory responses.

In respect to epigenetic changes, iron deficiency is known to alter key metabolic and epigenetic pathways, particularly of neural cells, including the phosphorylation of proteins involved in iron sequestration, glutamate metabolism, and histone methylation (185–187); also, liver hepcidin expression, as well as the liver BMP-SMAD signaling pathway, is suppressed by microRNA (188, 189); however, no significant differences in circulating microRNAs between iron-deficient and -replete persons have been observed (190), although some seem to participate in iron homeostatic events (191).

Vitamin A/D and iron homeostasis are very closely linked, making it difficult to distinguish the individual contributions of each micronutrient. For example, vitamin A promotes regulatory T cells (192) but also impacts macrophages and is a known contributor for iron mobilization (193) and—uptake (194), whereas deficiencies of both iron and vitamin A are associated with inflammation (195, 196).

Similarly, iron is also essential for vitamin D synthesis (197), so that people with iron deficiency usually have vitamin D deficiency too (198, 199), which likewise is linked to inflammation (200).

### Preventive Diets

Regardless of the inadequate exposure of atopic individuals with people, animals, and microbes, the “right diet” can also prevent or alleviate allergic disease. The 2021 GINA (371) guideline recommends micronutrient intake in the form of fruits and vegetables not only to prevent asthma but also to improve asthma control and reduce the risk of exacerbation (Evidence A) (201). Among foods, milk and, here, in particular, the whey protein content appears to reduce the risk of atopy (atopic dermatitis, rhinitis, asthma) (202–204), and this association has been shown, especially for drinking unprocessed raw milk. Indeed, even allergic children could tolerate raw milk better than pasteurized shop milk, showing less allergic symptoms upon drinking raw milk in a human pilot study (205). The atopy preventive effect of milk correlates with the amount of whey proteins present in the milk (206, 207) and is lost by thermal treatment (204, 208).

The whey protein content in the milk is highest in summer when the animals are kept on pastures and is lower in winter (209, 210). Grazing also strongly affects the iron as well as polyphenol content in milk, which has, indeed, higher antioxidant properties than vitamin C or E (211). The polyphenol content in milk depends on the forage composition and ranges from 3.7 to 35.8 g per-liter milk (212, 213), whereas reported iron concentrations vary from 57 μg (214) to 1,500 μg per liter (215), which correspond to roughly 1–26 μM iron per-liter milk.

Due to the loss of the heat-sensitive protective factors in whey, the ultra-high temperature UHT milk usually offered today does not prevent atopy. In this regard, it is remarkable that the main component of the whey is the heat-sensitive beta-lactoglobulin (BLG) (216) with constitutes 50–60% of all whey proteins, from which we show that it has a tolerogenic effect when loaded with micronutrients.

BLG is a known binder of many polyphenols [catechins (217, 218)], quercetin (219, 220), luteolin (221), rutin (220), etc., which increases the anti-oxidant activity of BLG (218, 222, 223) and leads to enhanced intestinal uptake of these polyphenols (224). Concurrently, depletion of BLG reduces the antioxidant activities of milk by 50%, and, also, heating (that destroys BLG) reduces the antioxidant activity (225, 226), while purified BLG is only considered a mild antioxidant (225).

Similarly, there are numerous reports showing the iron-binding abilities of BLG (222, 224, 227, 228) as the major component in whey (229) improve iron absorption (230–233).

Milk processing such as pasteurization has been shown to cause aggregation of whey proteins (216) to impair the ligand-binding capacity of BLG—shown with ligands such as retinol and palmitic acid (234), while, at the same time, its antigenicity increases (234). Milk processing has also been described to decrease copper and iron content (235) in milk.

## Epidemiology and Clinical Evidence of Iron Deficiency in Atopic Diseases

With regard to iron deficiency and atopic diseases, large epidemiology consistently demonstrated that children with allergies have an up to eight-fold greater risk of developing iron deficiency anemia than children without allergies (112, 114). The greater anemic risk in allergic children is clinically relevant as iron deficiency during the years of growth not only causes fatigue and anemia but also affects the small intestinal function and cognitive development (attention, sensory perception, emotions, intelligence). Physicians caring for children with atopic diseases should clarify in their current practice whether fatigue is due to sleep loss caused by atopic dermatitis or asthma or whether an undiagnosed anemia is present.

Iron deficiency can be “inherited” as the nutritional state of the mother is passed to the child. As such, the iron status of pregnant women already predetermines the later allergy risk of children. Several studies demonstrated that a good iron status of the expectant mothers lowered the risk of children of developing atopic dermatitis or asthma (172, 176, 236, 237). Low maternal hemoglobin levels are also associated with increased IgE antibody levels and lower lung volume in the child. Higher maternal transferrin concentrations during pregnancy, reflecting a lower iron status, were associated with an increased risk of a child's physician-diagnosed inhalant allergy (238). In an Italian study, supplementing mothers with iron and folic acid during their pregnancy compared to women without nutrient supplementation reduced the risk of their children developing atopic dermatitis by the age of 6 years by 80% (176). An inverse association was also illustrated between cord blood iron levels (173) right after delivery and the development of atopic urticaria, infantile eosinophilia, and wheeze at 4 years of age (172, 173).

Even in adults, the anemia risk is pertained in allergic individuals. A Korean study analyzing health insurance records from the health care system revealed that men with allergies had a 3.5-fold higher risk of being anemic than non-allergic men, while, in women, this difference was only about half as large (115). A possible explanation for this gender discrepancy could be the natural fluctuations in women's iron status, which often change due to menstrual cycles, pregnancies, and contraceptive methods (copper IUD), as well as due to the general greater tendency for iron deficiency in women to be left untreated, even in the absence of allergies.

By the same token, patients with anemic diseases are also more likely to develop atopic diseases and asthma. Elevated IgE is a common phenomenon observed in anemic patients, which is not related to parasitic infestations (239). Patients with chronic, even life-threatening anemia as with beta-thalassemia major (Cooley's anemia)—having impaired hemoglobin synthesis, which is often accompanied by enlarged spleens, livers and hearts—are more likely to have atopic diseases (240, 241) and suffer from asthma (241–244). Similarly, also subjects with atopic dermatitis have a greater risk to suffer from coronary heart disease, angina, peripheral artery disease, and anemia (245).

Summing up, the studies provide evidence that, indeed, atopy and iron deficiency are interconnected, making anemia more common in allergic people than in non-allergic individuals.

## Immune Cells Under Iron-Deficient Conditions

### Neutrophils, Natural Killer Cells, and Macrophages—Lower ROS Formation, Despite Increased Activity

Neutrophils, monocytes/macrophages (246, 247) and NK cells (248) use iron to combat pathogens. During intracellular infection, they release iron-loaded lactoferrin into their phagocytic vacuoles where ferrous iron functions as a catalyst of the Haber-Weiss reaction, generating reactive oxygen species (ROS) (249). Hence, under iron-deficient conditions, ROS formation and microbicidal killing are impaired.

As macrophages also are the principal cells for iron distribution, iron-deficient conditions hamper their iron-distribution capability, shifting the macrophage toward a more pro-inflammatory phenotype. Consequently, nutritional iron deficiency has been implicated in low-grad inflammation (250) and shifting of monocytes to a more inflammatory state in children (251) and infants (252) (Figure 2).

### Lymphocytes–Survival Advantage for Th2 Cells

An important aspect of iron deficiency is that the decrease in red blood cells is often accompanied by an increase of the white blood cell population, in which particularly the lymphocytic population is significantly increased (253). Within the lymphocytes, however, particularly CD4+ cells and the CD4/CD8 ratio is reduced (253, 254).

Iron chelation inhibits T cell proliferation, as T cell activation leads to expression of TfR1 for iron uptake. As such, iron chelation partake in apoptosis induction of proliferating, activated T-lymphocytes, but not of resting peripheral blood lymphocytes or granulocytes (255). Besides iron-uptake *via* transferrin, also, active uptake of oligomeric ferric citrate has been reported for T cells (256, 257). T lymphocytes also actively modulate the NTBI pool by uptake and export, with T cell deficiency associated with iron accumulation in the liver and pancreas (258).

The acidity of lysosomes also seems to partake in iron homeostasis and cell proliferation. Under lysosomal pH augmentation, cellular iron *via* TfR1 is impaired, decreasing cellular viability and proliferation, whereas iron supplementation by augmenting the NTBI pool bypasses the need for functional and acidic lysosomes and rescues cellular viability and proliferation in T cells (259).

In regard, to T cell subtypes, particularly, inflammation-associated Th1 cells are sensitive to iron-deficient conditions (260) as iron regulates the IFN-gamma/STAT1 signaling pathway (261).

Iron import into T cells seems also to affect T cell polarization, as import of iron *via* iron-siderophore-laden LCN2 has been demonstrated to suppress TH17 polarization in a vasculitis model (262).

In contrast, patients with iron overload have relative lower numbers of CD3 + T cells, while their percentage of regulatory T (Treg) cells and the ratio of CD4/CD8 seemed increased (263).

**Figure 3:**
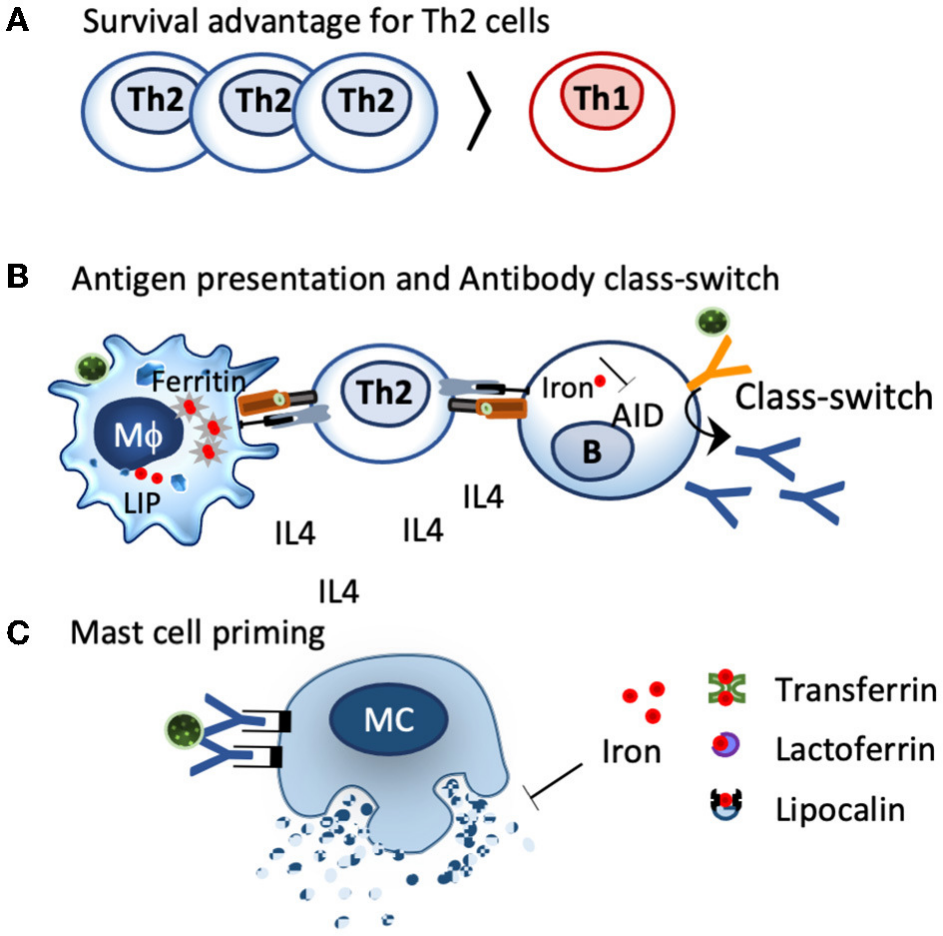
Impact of iron deficiency on immune cells. **(A)**. Th2 cells characterized by IL4 secretion have a greater chelatable iron pool compared to Th1 cells and have a survival advantage under iron-deficient conditions. **(B)**. Iron-deficient conditions modulate iron handling in macrophages and shift them towards a more activated, inflammatory status, which facilates antigen presentation. The activation-induced cytidine deaminase (AID), an enzyme responsible for class switch and affinity maturation, is repressed by iron. Iron-deficient conditions favor AID activation and class switch. **(C)** Local iron deprivation induces mast cell degranulation, whereas iron repletion by transferrin, lactoferrin, and lipocalins suppresses their activation.

Th2 clones exhibit larger chelatable iron pools than Th1 clones and are less affected by deferoxamine treatment or TfR1 blocking (264), resulting in a survival advantage of Th2 cells under iron-deficient conditions (260, 265, 266) (Figure 3). Consequently, iron deficiency prones the system toward Th2 (267), induces splenomegaly in mice (268), and induces increased IL-4 secretion in the supernatants of anti–CD3-treated splenocytes compared to controls (268).

Similarly, also in humans, iron deficiency *per se* generates a Th2 environment. In the seminal African study, which examined the immune status of children with or without iron deficiency, a marked elevation of the Th2 mediator interleukin 4 was also seen in children with iron deficiency, but not in iron-repleted children (269).

As such, under iron-deficient conditions, a Th2 environment is evidently created, which is the basic prerequisite for allergic sensitization (Figure 3).

### B Cells—Promotion of Antibody Class Switch and Affinity Maturation

Iron deficiency also affects antibody-producing B cells, as the enzyme responsible for antibody class switching and affinity maturation, the activation-induced cytidine deaminase, AID, is activated under iron-deficient conditions, while ferrous iron specifically inhibits this enzyme (270). In line, a lack of iron impairs in B cells adequate transfer of ferrous iron to the protoporphyrin IX in the mitochondria, thereby hampering heme synthesis and maintaining Bach2 activation (271), an essential transcription factor not only for class switching and affinity maturation but also an important regulator for T reg differentiation and the macrophage function (272).

In line, iron fortification of Vietnamese school children, but not deworming strategies, significantly improved hemoglobin, serum ferritin, and led to a significant decrease in the measured IgE-levels (239), with another study also reporting a decline in antibodies upon iron fortification in women (273). In contrast, decreased hemoglobin levels due to autoimmune hemolytic anemia, in which antibodies attack red blood cells (274), or because of infections (275) such as plasmodium falciparum malaria, digesting hemoglobin of the red blood cells (leading to anemia), are correlated with increased IgE-levels and severity (276).

The corollary of iron deficiency is, therefore, an antibody class switch toward IgE as iron deficiency simultaneously promotes a Th2 environment (Figure 3).

### Mast Cells—Ready to Burst

Mast cells, the main contributor for immediate allergic reactions, are particularly sensitive to iron deprivation. In these cells, intradermal application of the iron binder desferrioxamine, an iron chelator used in the clinics against iron overload, depletes the tissue and the resident mast cells of iron, resulting in histamine release and wheal formation (277). The iron binder is so effective that there have been endeavors to use the iron binder desferrioxamine instead of histamine as a positive control in skin tests. Reversely, iron delivery through transferrin, lactoferrin, or even iron-loaded beta-lactoglobulin (holoBLG) inhibits mast cell activation (12, 278–281) (Figure 3).

Interestingly, mast cells may also be involved in Th2-associated alopecia with an iron-restricted diet, resulting in hair loss in a murine model using IL10-deficient mice (282).

All in all, the degree of iron under- or oversupply seems to contribute directly to the reactivity of mast cells and, therefore, also on the symptom burden of allergic sufferers.

## Sequestration Strategies and Defense Mechanisms in Microbes and Plants

### Common Concepts in Bacteria and Fungi and Plants

Most bacteria and fungi require iron for their growth. In contrast to humans, in which iron is stored and transported predominantly within proteins, a very large pool of iron is present in bacteria (283) and fungi (284) in chelated form by low molecular compounds, with iron stored mainly in vacuoles and not within ferritin. Also, plants store iron in vacuoles and ferritin, although the distribution here varies with the type and development stage of the plant.

### Bacterial and Fungal Iron Acquisition Strategy

Bacteria and fungi such as Alternaria alternata thus usually have two types of siderophores: internal siderophores, such as fungal ferricrocin (285), and siderophores that are excreted such as coprogen for acquisition of environmental iron. Intracellular siderophores have been described to serve for iron storage and being involved in sporulation. In contrast, bacteria and fungi use exogenous siderophores, but also xenosiderophores, synthesized by other microorganisms, to acquire environmental iron as some microorganisms do not produce siderophores (286). The feeding with xenosiderophores is a widely used approach in bioassays in order to demonstrate their growth-promoting activity, and cross feeding is a widely observed feature of the microbial world (287) but also seems to extend to the host. Commensal bacteria such as Bacteroides fragilis have been reported to contribute to iron homeostasis of macrophage and be capable to modulate the immune response of macrophage (288). Siderophores may contribute thus in the nutritional provision of iron; in some cases, also binding to other metals such as copper, manganese, and zinc has been described, not only to support the microbial community, but that of the host too.

Indication for that exists in murine models in which the use of broad-spectrum antibiotics resulted in anemia and an altered immune homeostasis with diminished granulocytes and B cells (289), with fecal microbiota transfer partly reverting the hematopoietic changes (290). Antibiotic treatment also aggravated atopic dermatitis in a murine model (291, 292). In line, it is well established that individuals with atopic diseases (rhinitis, asthma, dermatitis, food allergy) have a reduced microbial (fungal and bacterial) diversity (108, 293–303), which may result in a diminished nutritional support by the commensal microbiota. The microbiota strongly manipulates the immune system. The composition and localization of the commensal microbiota in allergics may thus directly impact the homeostatic iron status of the host, but more studies here need to be done.

Bacteria use numerous iron uptake pathways, which include iron uptake from transferrin, ferritin, lactoferrin, siderophores, or heme. All of these uptake pathways require an active transport, although not all bacteria have all systems; e.g., *Listeria monocytogenes*, a facultative intracellular pathogen, can acquire iron through transferrin, lactoferrin, ferritin, and hemoglobin, but does not secrete any siderophores. Rather, it can use several hydroxamate (ferrichrome, ferrichrome A and ferrioxamine B) and catecholate (enterobactin and corynebactin) siderophores from other organisms, and it can use additional iron-binding compounds, such as hosts' catecholamines (304), gram-negative bacteria Neisseria spp., can acquire ferric iron directly from lactoferrin and serum transferrin *via* the TbpA/TbpB receptor (305, 306), and many bacteria exploit heme iron as a nutritional source (307) by secreting extracellular heme-binding proteins such as HasA (gram negative) and NEAT (gram positive) hemophores that either recognize heme and/or the host hemoproteins, such as hemoglobin, hemoglobin–haptoglobin and heme-hemopexin *via* HxuA hemophores (306, 308) to sequester and translocate iron into their cytoplasm (309).

### Iron Chelators: Siderophores and Flavonoids

Animals and humans provide a particularly low-iron habitat for bacteria and fungi. Consequently, siderophore production and access do play crucial roles in determining the course of an infection.

**Figure 4:**
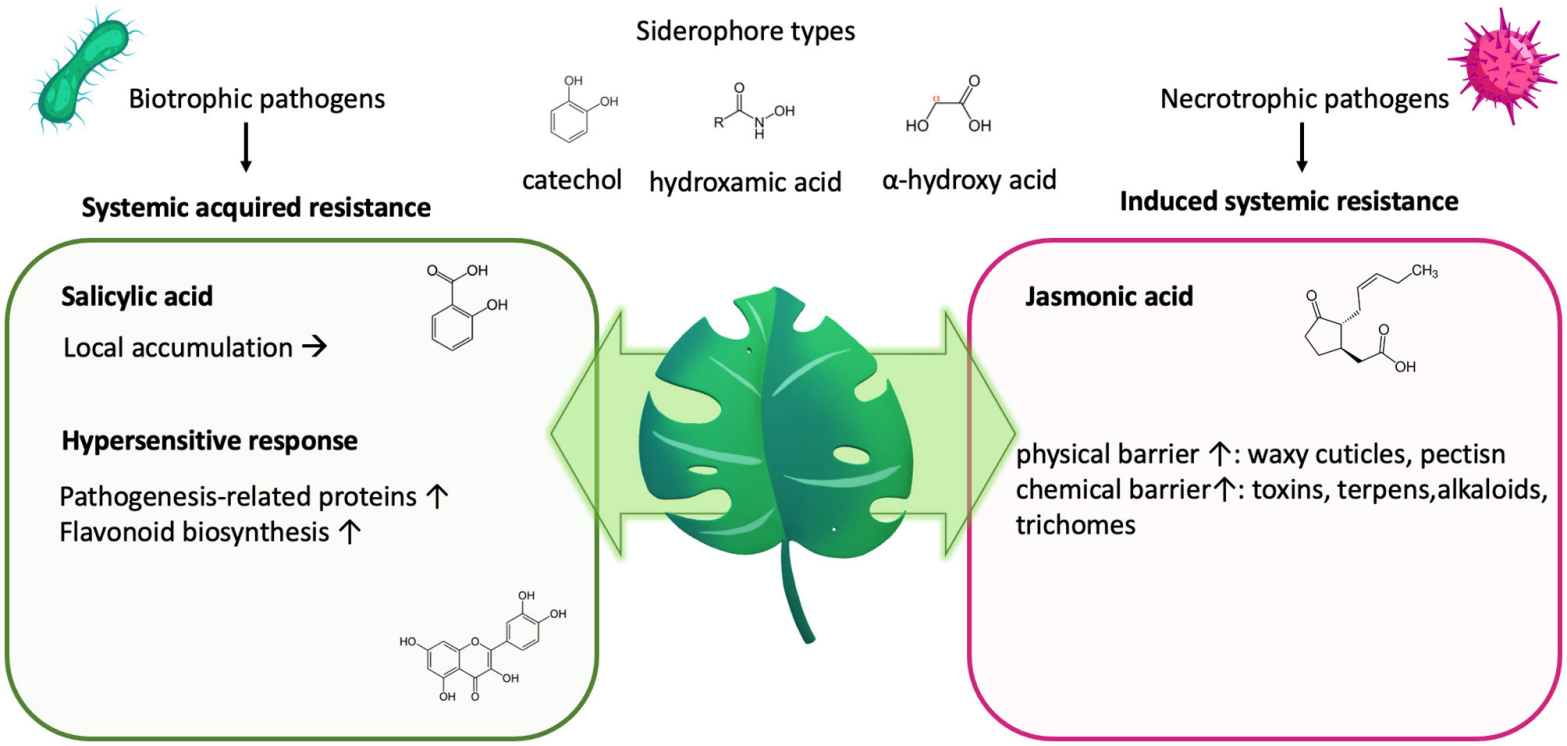
Plant defense and nutrition. Plants will impede biotrophic pathogens, releasing siderophores to sequester iron by initiating a local “hypersensitive response” as part of their “systemic acquired resistance.” This activates the salicylic acid, leading to its accumulation on site and the synthesis of pathogenesis-related proteins and polyphenols/flavonoids. Both can impede nutrient deprivation by the invading pathogen. In contrast, induced systemic resistance counter regulates the systemic-acquired resistance but leads to fortification of the physical and chemical barrier.

Siderophores are ferric iron–chelating molecules with very high ferric-ion association constants (10^20^-10^49^ M^−1^), which effectively remove iron from the host's iron–protein complexes. They are usually classified by their chemical moieties used to chelate the ferric iron, which are catechol-, hydroxamate or α-hydroxycarboxylate- moieties (Figure 4), but also mixed forms exist (162). Dependent on the moiety and the rest of the structure as well as salt type, ionic strength and temperature, there exist optimal pH-ranges for the respected siderophore types, with ferric iron usually complexed in an octahedral hexadental arrangement. Although dependent on the specific conditions, tris- and bis-catechol -ferric complexes possess some of the highest known stability constants of metal-ligand chelates, with the pH required to establish these bis- and tris-complexes being typically reported to be above pH 7 (310). In contrast, hydroxamates (311) usually have a wide roptimal pH range from 4 to 9, and described optimal chelation conditions for alpha-hydroxycarboxylates usually lie within the pH of 5–7 (66).

Generally, siderophore production is downregulated at low pH and upregulated with high pH (312).

Siderophores anti-oxidative and anti-inflammatory properties are widely acknowledged (313) as they can impede ROS formation.

As the biosynthesis of siderophores needs energy in form of carbon sources and ATP, it determines with the microbial growth rate, which kind of population will colonize a low-iron habitat. Microorganisms that continuously produce siderophores are unknown in nature. Similarly, siderophore production in fungi starts just after germination from conidiospores and are contained in the spore wall material, which is released during germination (314).

As secondary metabolites siderophores are generally defined for not being directly involved in the growth, development, and reproduction of the organisms, but mediate ecological interactions, which may produce a selective advantage for the microbes or plants. As such, microbial siderophores usually belong to the class of nonribosomal peptides (315) and/or polyketides (316), from which a number of very powerful medicinal products are known for, ranging from antibiotics (e.g., vancomycin) to immunosuppressive drugs, such as ciclosporin.

Similarly, many fruits and plants synthesize phenolics/polyphenols/flavonoids with described anti-oxidative and anti-inflammatory attributes, that—as their microbial counterpart—are categorized as secondary metabolites and have a very high affinity to iron due to the presence of catechol structures. For flavonoids, the reported complex stability constants for catechol are 43.7; for quercetin 44.2; and for catechine 47.4 (67) and thus comparable to the iron affinity of microbial siderophores, with the strongest known catechol-siderophore enterobactin having a complex stability constant of 49 at physiological pH (317).

Of note, many flavonoids-binding iron such as luteolin (318), apigenin, quercetin (319), catechin, rutin, naringenin, fisetin (320), and epicatechin have been attributed an anti-allergic activity *in vitro* and in *in vivo* models (321, 322). With a double-blind, placebo-controlled study using topical cream containing vitamin E, epigallocatechin gallate and grape seed procyanidins improving atopic dermatitis (323), and O-methylated catechins reducing symptoms of Japanese cedar pollinosis (324).

### Plant Defense and Iron Availability

Iron availability is dictated by the soil redox potential and pH. In soils that are aerobic or of higher pH, iron is readily oxidized, and is predominately in the form of insoluble ferric oxides. At lower pH, the ferric iron is freed from the oxide and becomes more available for uptake by roots. Because 30% of the world's cropland is too alkaline for optimal plant growth (e.g., calcareous soils in which the addition of lime increases the pH), graminaceous plants (grasses, cereals, and rice) secrete phytosiderophores (e.g., mugeneic acid), but also chemical compounds with catechol moieties have been described such as fraxetin (325), which are released into the soil to sequester iron (326).

Importantly, similarly than in the mammalian system, iron deficiency alone has been demonstrated to be enough to prime the plant immune response (327) and activate flavonoid (328, 329) and phytosiderophore synthesis (330).

Plants will impede pathogens by increasing their resistance *via* “induced systemic resistance” (Figure 4), which involves the synthesis of jasmonic acid and ethylene and leads to an increase of the physical or chemical barrier of the host plant (331). Simultaneously, upon infection, also, “systemic acquired resistance “is initiated, which is analogous to our innate immune system and mediated by synthesis of salicylic acid, leading to its accumulation, but also to the transcription of a wide range of “pathogenesis-related” proteins (332–334) as well as the synthesis of flavonoids (328, 335, 336) (Figure 4). Both pathways counter regulate each other, with salicylic acid inhibiting jasmonic acid signaling (336).

In response to pathogens, the salicylic acid pathway elicits a rapid local reaction or “hypersensitive response” to limit the area of infection for biotrophic pathogens, which require living tissue to gain nutrients. In the case of necrotrophic pathogens, hypersensitive response might even be beneficial to the pathogen, as they require dead plant cells to obtain nutrients.

Strikingly, many major allergens are derived from these pathogenesis-related protein families that are induced by the plants to prevent nutritional deprivation (337, 338).

Also, beneficial root-associated mutualistic microbes living in the rhizosphere, like bacteria and fungi, besides impacting on plant nutrition and growth, can further boost plant defenses, rendering the entire plant more resistant to pathogens (339). These beneficial microbes secrete siderophores to facilitate plant iron acquisition with ectorhizosphere and rhizoplane bacteria described to release predominantly hydroxamate-type siderophores, whereas endophytic bacteria rather producing catechol-type siderophores (340) for plant uptake. Interestingly, several different bacterial genera, especially in plant-growth-promoting rhizobacteria, synthesize salicylic acid, the key compound of the systemic acquired resistance in plants, to ultimately incorporate them into catechol-based siderophores (341).

Importantly, although a mutualistic relationship between hosts and microbial siderophores exists, at the same time, not only a competition between excreted siderophores for the metal but also for capturing these iron-siderophore complexes is always prevalent.

## Allergens or Tolerogens: the Role of Proteins Carrying Micronutrients

Only a few protein families are capable to become allergens under physiological conditions; thus, virtually, all major allergens of animal origin belong to the lipocalin family, specifically in the lipocalin subfamily of “retinoic acid-binding proteins” (11, 342) and a considerable part of the major respiratory allergens of plant origin belongs to the pathogenesis-related-10 (PR-10) protein family^10^ or originates from the prolamin (2S albumin, lipid-binding proteins, LTPs) and cupin (7S, 11S) superfamilies (216, 343).

Apart from belonging either to animal or plant allergen families, they do have several features in common with the most essential one, that these proteins belong to the innate defense system in the respected animals/plants. They, therefore, possess an inherent affinity to our immune system, and their uptake occurs mostly receptor mediated and *via* the lymphatic system. The described allergen families have “pockets” in which they can very effectively bind and transport micronutrients, such as iron complexes, fatty acids (344), flavonoids (217–221) or vitamins (10, 281, 345–348). In this way, they can deprive pathogens of nutrients or, conversely, provide nutrients to the immune cells.

As such, many major allergens are capable to bind to flavonoids with known iron-binding capacity, making them nutrient binders. Consequently, the natural ligand of the pathogenesis-related PR-10 proteins major birch pollen allergen Bet v 1 has been identified as quercetin-3-O-sophoroside (349); for the major hazelnut allergen Cor a 1, being quercetin-3-O-(2″-O-β-D-glucopyranosyl)-β-D-galactopyranoside (350), and also Fra a 1 and Fra a 3 have been crystalized with catechin ligands (351). Also, other major allergens from peanuts have been well investigated with Ara h 2 and Ara h 6, belonging to the 2S family, binding to the flavonoid epigallocatechin-3-gallate (352), Ara h8 binding to quercetin, (353) as well as epicatechin (354) and Ara h 1 from the 7S family, forming large complexes by binding to proanthocyanidins, which are oligomers, consisting of catechin and epicatechin and their gallic acid esters (355).

Mammalian lipocalin allergens closely resemble endogenous human lipocalin proteins, such as Lipocalin-2, LCN2 (11, 157), a natural acute phase defense proteins that binds environmental iron and can deliver this iron directly and a receptor-mediated to immune cells (157, 162). They are usually excreted and thus are found in the dander, urine, fur, and saliva of animals (356). LCN2 is involved in numerous iron-dependent processes of the innate immune arm and is also critical to renal development. Iron transport by lipocalins requires the presence of a siderophore, since lipocalins usually have no measurable affinity for iron alone (357). Consequently, LCN2 binds only to iron chelated by siderophores, thereby being also microbicidal. Simultaneously, it acts as an immune regulator as the iron-containing form of LCN2 (holoLCN2) increases the intracellular iron content of macrophages, while the iron-free form decreases the intracellular iron content (358). Thus, raising of the labile iron pool content by iron-loaded LCN2 form promotes the development of anti-inflammatory cells (359–361), while the lowering of their intracellular iron content causes their activation. Importantly, LCN2 is able to activate or suppress the immune cells—dependent on the nutritional supply it provides.

Due to its resemblance to lipocalin 2, mammalian lipocalins, such as the bovine beta-lactoglobulin BLG, are similarly taken up *via* the lymphatic system (216, 362); in a receptor-mediated fashion and *via* this route, their ligands will predominantly transport to the residing immune cells. It can even reach the lactal system of nursing mothers and serves as a marker for maternal dietary proteins in breast milk as it is not naturally present in human milk (363). In a series of studies exploiting the lymphatic pathway for targeted micronutritional supply of iron (10, 12, 281), zinc (281), and vitamins (346) by BLG, we provided evidence that micronutrients were transported to immune cells, and that this nutritional supply was accompanied with the establishment of immune resilience in an allergen-independent fashion (12, 348) in a prophylactic setting, as well as in already sensitized mice, this leads to a significant reduction of the symptom burden upon allergen challenge (281).

Our studies, but also these of others (364, 365), have demonstrated that, in the absence of micronutrients, particularly of iron, proteins of the innate defense arm in mammals and plants in their apo-(empty) form are able to elicit a Th2 response *in vitro* and *in vivo* (10, 12, 346, 347) as an encounter of these proteins in an “empty” form with our immune system enables them to locally deplete these cells from iron or vitamins, thereby triggering a danger signal and evoking an immune response. In contrast, when these proteins carry micronutrients and are present as holo-(loaded) proteins, they contribute to the nutritional balance of the immune cell and actively contribute to tolerance development (10, 12, 162, 281, 345–348).

Thus, upon contact with the holo-proteins, the immune nutritional balance is not disturbed, enabling the establishment of immune resilience (12), which protects against atopy.

In situations of infections or inflammation, which requires an increased micronutritional supply, or when nutritional deficiencies are already prevalent, apo-proteins can bind to micronutrients, further aggravating the micronutritional deficiency present in these cells, which not only activates these immune cells but also results that exogenous innate defense proteins are recognized as a threat and turn into allergens.

## Clinical Studies: Balancing Micronutrient Requirements as a Strategy to Ameliorate Allergic Diseases

Based on the preclinical studies, we sought clinical translation of our research efforts and combined the whey protein BLG with catechines, iron, zinc, and vitamin A into a lozenge (holoBLG lozenge) to be used as a food for special medical purposes (FSMP). The ultimate objective was to investigate in clinical studies whether, indeed, the targeted transport of micronutrients to immune cells by holoBLG was effective and could have an influence on immune cell reactivity and the allergic symptom load in allergic individuals.

Of note, the amount of iron included in the lozenge is with <1 mg/lozenge rather low, and, therefore, the lozenge cannot be considered as an iron supplement *per se*, but it does contain iron in a form that enables transport by BLG *via* the lymph and is roughly equivalent to the estimated daily iron requirement of human leukocytes.

In the 2019 and 2020 conducted double-blind, placebo-controlled clinical trial with women allergic to birch and/or grass pollen allergy, 6-month supplementation with holo-BLG lozenge resulted in a total nasal symptom score (TNSS) improvement after nasal provocation by 42% after, compared with 13% in the placebo group. The combined symptom-medication score, considered the gold standard of allergen immunotherapy, (366) was in the group, taking the holoBLG lozenges 45% lower in the birch pollen peak season and 40% lower in the grass pollen season compared to the placebo-supplemented study arm. Additionally, blood values improved, and peripheral blood monocytic cells had, compared to the monocytes of the placebo arm, a significant higher labile iron pool (12, 347, 367, 368).

Another clinical study with house dust mite allergic patients was also conducted in 2020, in which the symptoms were objectively assessed and recorded in an allergen exposure chamber before and after 3 months of holoBLG supplementation. Here, holoBLG supplementation resulted in a 60% reduction of the TNSS (369). Moreover, a long-lasting effect was apparent, as even 7 to 8 months later these patients had lower total symptom score and a perceived higher well-being on re-exposure in the allergen exposure chamber, indicating a long-lasting nature of the induced immune resilience (370).

It has to be emphasized that in both atopic cohorts, dietary application of the holoBLG lozenge containing micronutrients, that are dedicated for the immune cell compartments, ameliorated allergic symptoms in a completely allergen-independent manner.

Further studies are currently being conducted with cat allergic patients to investigate in other atopic cohorts, whether compensating micronutritional deficiencies in the immune cell compartments is a further causal strategy to support immune resilience in an allergen-independent manner.

## Discussion

Iron is a trace element essential for nearly every organism and needed for oxygen transport, cellular respiration, but also contributing in immune regulation. Its access is tightly controlled due to its high affinity for oxygen, requiring that iron always has to be present in a complexed and/or protein-bound form; otherwise, reactive oxygen species are generated with detrimental effects.

Here, we collected evidences that functional iron deficiency not only promotes allergy development but also increases the clinical symptom burden in allergic patients.

Atopic individuals lack—besides Vitamin A and D—iron, which profoundly affects our immune system as deficiencies here render our cells hyper-sensitive.

The dual role of macrophages as the central hub for iron handling but also as a major contributor in immunity has the consequence that iron deficiency directly impacts these cells and shifts them under iron poor conditions to a more inflammatory phenotype.

Iron deficiency is sufficient to create a Th2-milieu to favor affinity maturation and antibody class switching and to prime mast cells for degranulation. Consequently, iron deficiency sets the whole body on alert.

Although this a very desired response to infections, it also turns, otherwise, harmless proteins to allergens.

Indeed, comparing the defense system in the plant with ours is particularly revealing as, here, it becomes apparent how intricate nutrition and defense are intertwined and that stealing and sharing often go hand in hand. On the one hand, the biotrophic pathogen needs its nutrients from the host and secretes anti-inflammatory siderophores, and its attack is being counteracted by pathogenesis-related proteins, hindering nutritional retrieval. On the other hand, microbes synthesize their siderophores from salicylic acid and share the nutrients bound by siderophores with their host, thereby promoting the growth and health of the plant. Similarly, interactions can be assumed in humans with uptake of flavonoids being well-documented, but also the commensal microbial communities will participate in the nutritional provision of the human host, with the secondary metabolites of some commensal bacteria already known to be capable to modulate iron handling in human macrophages.

Exactly, these ecological interactions seem lacking in individuals with atopy, with the microbial communities either not able or not sharing their precious micronutrients with the host but also the individuals with atopy secreting less lipocalin and other innate proteins capable to capture this precious siderophore-complexed iron. Due to the precarious nutritional status, the antigen-presenting cells of atopic persons are also much more sensitive to potential “nutrient” thieves in the form of allergens. In contrast, encountering these allergens with micronutrients seems to turn them into friends and tolerogens.

Once functional iron deficiency is established, dietary iron absorption is hindered by hepcidin, resulting that those persons with functional-iron deficiency (and inflammation) are in the vicious cycle, in which they need more iron but have to exploit different nutritional approaches to compensate their iron requirements, as, otherwise, their immune systems remain hyperactive. Here, evidence is given that one dietary approach is by the lymphatic route using the whey protein beta-lactoglobulin as a carrier for micronutrients.

Our preclinical as well as clinical studies demonstrated that iron can be selectively transported to the myeloid cells through holoBLG, thereby reestablishing immune resilience. Indeed, supplementation with holoBLG could simulate “the protective farm effect” as, also here, protection against allergies could be achieved in a completely allergen-independent manner.

To date, specific allergen immunotherapy is considered the only causative treatment option for ameliorating atopic diseases. However, providing immune cells with micronutrients shows a strikingly similar efficacy, in a completely allergen-independent manner. It emphasizes that micronutritional provision is another causative cure against allergies that should be included in the current practice.

## Author Contributions

The author confirms being the sole contributor of this work and has approved it for publication.

## Funding

This study was supportd by the Danube Allergy Research Cluster (DARC) project #08 to Erika Jensen-Jarolim, Karl Landsteiner University Krems, Austria.

## Conflict of Interest

The author declares inventorship of EP2894478 (Roth-Walter F et al. Method and means for diagnosing and treating allergy.) (applicant Biomedical International R+D GmbH, Vienna, Austria), the basis for the holoBLG lozenge. FR-W received research funding from Biomedical International R+D GmbH, Vienna, Austria, Bencard Allergie GmbH, Munich, Germany and Vienna, Austria, and Allergy Therapeutics, Worthing, UK. Moreover, she received lecture honoraria by FOMF, VAEM, Bencard Allergie GmbH, Munich, Germany and Vienna, Austria, and Allergy Therapeutics, Worthing, UK.

## Publisher's Note

All claims expressed in this article are solely those of the authors and do not necessarily represent those of their affiliated organizations, or those of the publisher, the editors and the reviewers. Any product that may be evaluated in this article, or claim that may be made by its manufacturer, is not guaranteed or endorsed by the publisher.
